# HIV pre-exposure prophylaxis and early antiretroviral treatment among female sex workers in South Africa: Results from a prospective observational demonstration project

**DOI:** 10.1371/journal.pmed.1002444

**Published:** 2017-11-21

**Authors:** Robyn Eakle, Gabriela B. Gomez, Niven Naicker, Rutendo Bothma, Judie Mbogua, Maria A. Cabrera Escobar, Elaine Saayman, Michelle Moorhouse, W. D. Francois Venter, Helen Rees, on behalf of the TAPS Demonstration Project Team

**Affiliations:** 1 Wits Reproductive Health and HIV Institute, University of the Witwatersrand, Johannesburg, South Africa; 2 Department of Global Health and Development, London School of Hygiene &Tropical Medicine, London, United Kingdom; 3 Department of Public Health Policy, London School of Hygiene &Tropical Medicine, London, United Kingdom; 4 Amsterdam Institute for Global Health and Development, Department of Global Health, Academic Medical Center, Amsterdam, The Netherlands; 5 Sediba Hope Medical Centre, Tshwane, South Africa; 6 Department of Clinical Research, London School of Hygiene &Tropical Medicine, London, United Kingdom; Desmond Tutu HIV Centre, SOUTH AFRICA

## Abstract

**Background:**

Operational research is required to design delivery of pre-exposure prophylaxis (PrEP) and early antiretroviral treatment (ART). This paper presents the primary analysis of programmatic data, as well as demographic, behavioural, and clinical data, from the TAPS Demonstration Project, which offered both interventions to female sex workers (FSWs) at 2 urban clinic sites in South Africa.

**Methods and findings:**

The TAPS study was conducted between 30 March 2015 and 30 June 2017, with the enrolment period ending on 31 July 2016. TAPS was a prospective observational cohort study with 2 groups receiving interventions delivered in existing service settings: (1) PrEP as part of combination prevention for HIV-negative FSWs and (2) early ART for HIV-positive FSWs. The main outcome was programme retention at 12 months of follow-up. Of the 947 FSWs initially seen in clinic, 692 were HIV tested. HIV prevalence was 49%. Among those returning to clinic after HIV testing and clinical screening, 93% of the women who were HIV-negative were confirmed as clinically eligible for PrEP (*n =* 224/241), and 41% (*n =* 110/270) of the women who were HIV-positive had CD4 counts within National Department of Health ART initiation guidelines at assessment. Of the remaining women who were HIV-positive, 93% were eligible for early ART (*n =* 148/160). From those eligible, 98% (*n =* 219/224) and 94% (*n =* 139/148) took up PrEP and early ART, respectively. At baseline, a substantial fraction of women had a steady partner, worked in brothels, and were born in Zimbabwe. Of those enrolled, 22% on PrEP (*n =* 49/219) and 60% on early ART (*n =* 83/139) were seen at 12 months; we observed high rates of loss to follow-up: 71% (*n =* 156/219) and 30% (*n =* 42/139) in the PrEP and early ART groups, respectively. Little change over time was reported in consistent condom use or the number of sexual partners in the last 7 days, with high levels of consistent condom use with clients and low use with steady partners in both study groups. There were no seroconversions on PrEP and 7 virological failures on early ART among women remaining in the study. Reported adherence to PrEP varied over time between 70% and 85%, whereas over 90% of participants reported taking pills daily while on early ART. Data on provider-side costs were also collected and analysed. The total cost of service delivery was approximately US$126 for PrEP and US$406 for early ART per person-year. The main limitations of this study include the lack of a control group, which was not included due to ethical considerations; clinical study requirements imposed when PrEP was not approved through the regulatory system, which could have affected uptake; and the timing of the implementation of a national sex worker HIV programme, which could have also affected uptake and retention.

**Conclusions:**

PrEP and early ART services can be implemented within FSW routine services in high prevalence, urban settings. We observed good uptake for both PrEP and early ART; however, retention rates for PrEP were low. Retention rates for early ART were similar to retention rates for the current standard of care. While the cost of the interventions was higher than previously published, there is potential for cost reduction at scale. The TAPS Demonstration Project results provided the basis for the first government PrEP and early ART guidelines and the rollout of the national sex worker HIV programme in South Africa.

## Abstract 2

In an observational study, Gabriela Gomez and colleagues report on provision of pre-exposure prophylaxis and antiretroviral treatment for female sex workers in South Africa.

## Author summary

**Why was this study done?:**

- Female sex workers are a vulnerable population at risk of HIV, and, in some areas of South Africa, nearly 3 out of 4 sex workers are already infected.
- Pre-exposure prophylaxis (PrEP) and early antiretroviral treatment (ART) are interventions being piloted by national programmes to test whether (and how) they can be successfully delivered in clinics and the public sector.
- We conducted a study in existing sex-worker-specific clinics to see if female sex workers would take up and use PrEP and early ART if these interventions were offered. We partnered with the South African government to align and incorporate our results into national HIV programming.

**What did the researchers do and find?:**

- We offered PrEP to HIV-negative female sex workers and early ART to HIV-positive female sex workers in 2 clinics located in Johannesburg and Pretoria in South Africa. Over time, we assessed how many women were contacted for the intervention, how many came to the clinic for HIV testing, how many were HIV-negative and -positive, and how many took up the interventions. We then monitored those who participated for at least 12 months to see whether they stayed in the programme and used PrEP or early ART.
- We found an HIV prevalence of 49% among the 692 women tested. Among those returning to clinic after HIV testing and clinical screening, 93% were confirmed eligible for PrEP, and 94% for early ART. From those eligible, 98% and 94% took up PrEP and early ART, respectively. At the end of the 12-month assessment, 22% remained on PrEP, while 60% remained on early ART, at our clinic sites. It is possible that some women moved to other sites without our knowledge, as other sites started offering PrEP and early ART.
- The drugs were safe to use with few side effects. We also found that women who stayed in the study reported taking their pills regularly. None of the women who remained in the study in the PrEP group got HIV, and very few women taking early ART had issues supressing the virus. The overall cost of the programme came to approximately US$126 for PrEP and $406 for early ART per person-year.

**What do these findings mean?:**

- These findings suggest that female sex workers may be interested in both PrEP and early ART and that the interventions work well when taken, but longer term commitment may be a challenge.
- These interventions have already been introduced by the South African government into sex worker programmes, including those for male, female, and transgender people.

## Introduction

Rates of new HIV infections remain high, especially in key populations in sub-Saharan Africa, necessitating new options for HIV prevention and treatment [1,2]. Mathematical and clinical studies suggest that a programme combining current HIV prevention options with oral pre-exposure prophylaxis (PrEP), i.e., giving antiretrovirals to uninfected individuals, and early antiretroviral treatment (ART), i.e., giving antiretrovirals to HIV-infected individuals irrespective of CD4 count, could make significant inroads on reducing new infections in key and general populations [3–7].

Following the transition of WHO treatment guidelines to recommending both PrEP and early ART, research has shifted focus to answering questions about implementation [5], including uptake, adherence, and retention in existing programmes [8]. Demonstration projects, open-label extension studies, and population-based implementation trials testing feasibility in ‘real world’ settings are ongoing or have been recently completed [9,10]. Most of the completed demonstration projects have focused on men who have sex with men (MSM) populations throughout the world (though primarily in the United States, South America, and Europe) [10]. One or two other projects have been completed among serodiscordant couples [11], and only one randomised control trial implementation study had been completed among female sex workers (FSWs) at the time of the writing of this report [12].

In South Africa, although sex work is still criminalised, FSWs have been prioritised for focused, tailored services in the previous and new National Department of Health (NDoH) national strategic plans for HIV, as well as a specialised plan for sex workers, as the population with the highest incidence and prevalence of HIV, especially in urban areas [13–15]. The national sex worker HIV programme includes support for demonstration projects to further the development of specialised services including PrEP and early ART.

The TAPS (Treatment And Prevention for female Sex workers) Demonstration Project, nested within the long-standing Sex Worker Programme (SWP) [16], was designed to support integration of oral PrEP, as part of a combination prevention approach, and early ART into existing HIV services in 2 urban settings, with specific aims to assess uptake, retention, and adherence among FSWs and to estimate the cost of this strategy. TAPS was the first demonstration project in South Africa to include PrEP and early ART for FSWs, and among the first few in sub-Saharan Africa as well. This paper presents the primary results.

## Methods

The TAPS Demonstration Project was a prospective observational cohort designed as a real-world implementation study to integrate PrEP, as part of a combination prevention approach, and early ART into existing services for evaluation. The protocol has been described in detail in a previous publication [17]. The TAPS Demonstration Project protocol was approved by the University of the Witwatersrand Human Research Ethics Committee (reference number: 140502) and the South African Medicines Control Council (reference number: 20140740). All participants completed a written informed consent process for the study consisting of a comprehensive consent form for the main study, as well as additional forms for laboratory sample storage and the qualitative study components. Costing studies were also performed after securing consent from clinic staff.

### Setting and intervention

TAPS was embedded within the SWP, operated by Wits Reproductive Health and HIV Institute, which is integrated into the South African public health clinical service. The SWP has been in existence since 1996 and is run by nurses, community health workers, and peer educators [16]. Systematic formative research conducted to support the design and in preparation for TAPS is presented elsewhere [18].

The 2 clinic sites were situated in inner-city Johannesburg and Pretoria. These clinics cater to an urban population of sex workers working in hotel brothels, on streets, and in other informal environments. The clinics provide standard-of-care primary healthcare including HIV testing services, male and female condom distribution, nurse-initiated and managed ART, tuberculosis (TB) screening, contraceptive provision, cervical cancer screening, clinical services for sexually transmitted infections (STIs) and minor ailments, psychosocial support, and referrals for pregnancy and other clinical and legal services.

Recruitment of FSWs took place in the clinics and in surrounding brothels, bars, and streets, relying on existing peer educator outreach services. Screening for participation was conducted in 2 steps. First, women were tested for HIV using a standard-of-care rapid test. This step included obtaining NDoH HIV testing service written consent and asking brief questions as to current pregnancy and whether they were a sex worker (e.g., they were asked to self-identify after peer recruitment in their places of work). Women were then either referred to other relevant care (if they were pregnant, were not a sex worker, or decided not to proceed) or they continued on to clinical screening for the TAPS study. This process always occurred on the same day to ensure that HIV testing and clinical assessments were aligned. The next phase of the screening process began by obtaining study-specific written consent, then continued with the assessment of clinical history, administration of demographic and behavioural questionnaires, TB and pregnancy screening, and blood draws for creatinine measurement, hepatitis B screening, and syphilis testing. HIV status confirmation with ELISA was done for HIV-positive participants at a private laboratory, where all the other samples were also analysed. Women were offered contraception, as well as advised of the need to use condoms to prevent HIV, STIs, and unwanted pregnancies.

After screening, participants were asked to return a week later, to allow for laboratory analyses to be completed, for potential enrolment into either PrEP for HIV-negative FSWs or early ART for HIV-positive FSWs. The definition of early ART has evolved in line with national guidelines. At the start of the study, the CD4 threshold for treatment initiation was 350 cells/ml; the threshold changed to 500 cells/ml, and then treatment initiation became independent of CD4 count during the course of the study. Since our purpose was to offer treatment to those not eligible for ART under current guidelines, TAPS transitioned with the national treatment initiation definition. Project milestones are provided in S1 Fig. Women were eligible for the study if they were age 18 years or over, were not pregnant at enrolment, did not have multidrug-resistant TB, were not participating in another clinical study, and had finished their regimen for post-exposure prophylaxis if they were taking it at the time of assessment. Those who became pregnant through the course of the study were given the option to remain in the study on current medication, discontinue use during the period of pregnancy and remain in the study, or transfer out completely to antenatal care. The first 2 options also included referrals to antenatal care.

Once initiated on PrEP or early ART, participants were scheduled for an initial 1-month check-in to assess safety and/or adherence issues, and then were scheduled for quarterly clinical testing and safety monitoring visits thereafter. All clinical monitoring followed South African national guidelines throughout the study (also detailed in the published protocol) [17], and all services were provided free of charge as per the public health clinic standard, including PrEP and early ART. Participants were given refills of 1–3 months depending on ability to safely store the products and desire to come to the clinic for refills. The importance of consistent and high adherence was emphasised for PrEP use; however, participants could cycle on and off medication during periods of lower risk as desired and remain in the study. Indeed, the PrEP intervention for HIV-negative women included not only the use of PrEP but also other available prevention options (such as condoms, continuous testing and counselling, and STI screening). Participants were offered short message service (SMS) reminders for clinic visits and positive messaging around adherence, health, and psychosocial issues.

For both groups, participants were asked about pill-taking habits using a motivational technique at each staff touch point: counselling, clinical, and pharmacy. Counselling followed the current standard of care for ART provision. Blood samples were taken for analysis of drug levels in PrEP participants, the results of which will be presented at a later date as they are not currently available. Adverse events were recorded per Good Clinical Practice guidelines and WHO recommended grading [19,20].

### Evaluation

Programmatic data to describe eligibility and uptake were collected in activity reports including the number of outreach contacts and appointments booked. Routinely collected demographic, behavioural, and clinical data were also analysed. Retention in care at 12 months was the primary outcome. Participants exited the analysis if they withdrew from the study or were lost to follow-up, defined as no contact over 2 consecutive quarterly clinic visits or not completing an exit visit at 12 months. Participants were considered retained in the programme if they had not withdrawn or been lost to follow-up during the 12-month period. We used an adapted cascade approach to measure uptake and retention through a series of touch points with participants. These points started during outreach and the enrolment process. Retention data are reported at each of the time points (3, 6, 9 and 12 months). Women who missed visits and returned later were included in the analysis. Data for those women who completed more than 12 months of follow-up are included in S3 Text.

Data were analysed on self-reported adherence and sexual behaviour, STI diagnoses, and occurrence of side effects. We also monitored any possible seroconversions on PrEP and virological failures on early ART. Qualitative research including in-depth interviews (with a selection of up to 10% of study participants), clinic observations, and group discussions with providers was also conducted and will be presented separately.

Costs were estimated from a healthcare provider perspective for each clinic using an ingredient approach. We included capital costs (equipment, buildings, non-recurrent training) as well as recurrent costs (personnel, supplies, management, and maintenance of buildings). Capital costs were annualised. Data collection activities included measurement of clinic space, completion of weekly timesheets by staff involved in the programme that detailed time per programme activity versus research activities, observations of practice detailing all clinical activities during participant visits, interviews with staff exploring time shared with other programmes, and review of expenditure and utilisation data with regards to the programme. Sources used in the valuation of all resources, and methods for allocation of shared resources, are detailed in Table A of S2 Text. We used both bottom-up and top-down approaches to calculate unit cost per visit. Economic costs were assigned to drugs as per procurement prices from NDoH for generics (US$4.8/month for PrEP; US$8.3/month for early ART); quantities of drugs dispensed per patient were sourced from clinical and pharmacy records. Services, activities, and healthcare providers involved in each type of visit are listed in Table B of S2 Text. We present unit costs for the different types of visits (outreach contact, testing session, enrolment, follow-up, and refill visits). Our unit costs per person-year for PrEP and early ART include enrolment, follow-up, and refill visits taking place during the first year of services and reflect the attendance patterns observed during the TAPS Demonstration Project. All prices were collected in local currency and are reported in 2015 US dollars. The average exchange rate used was US$1  =   R 11.6. All data were analysed in STATA 12 (StataCorp) and EXCEL 2016 (Microsoft).

A national programme including PrEP and early ART for FSWs was launched in June 2016, during the study, and may have influenced enrolment and retention. To test this, a stratified analysis of uptake and retention indicators is provided in S3 Text, comparing the cascade results among TAPS cohorts disaggregated by time of enrolment and follow-up period. We tested the null hypothesis that the 2 proportions are the same at each step of the cascade and report *p-*values for this testing. This analysis was the only additional analysis not prospectively defined.

## Results

### Outreach and uptake

**Fig 1:**
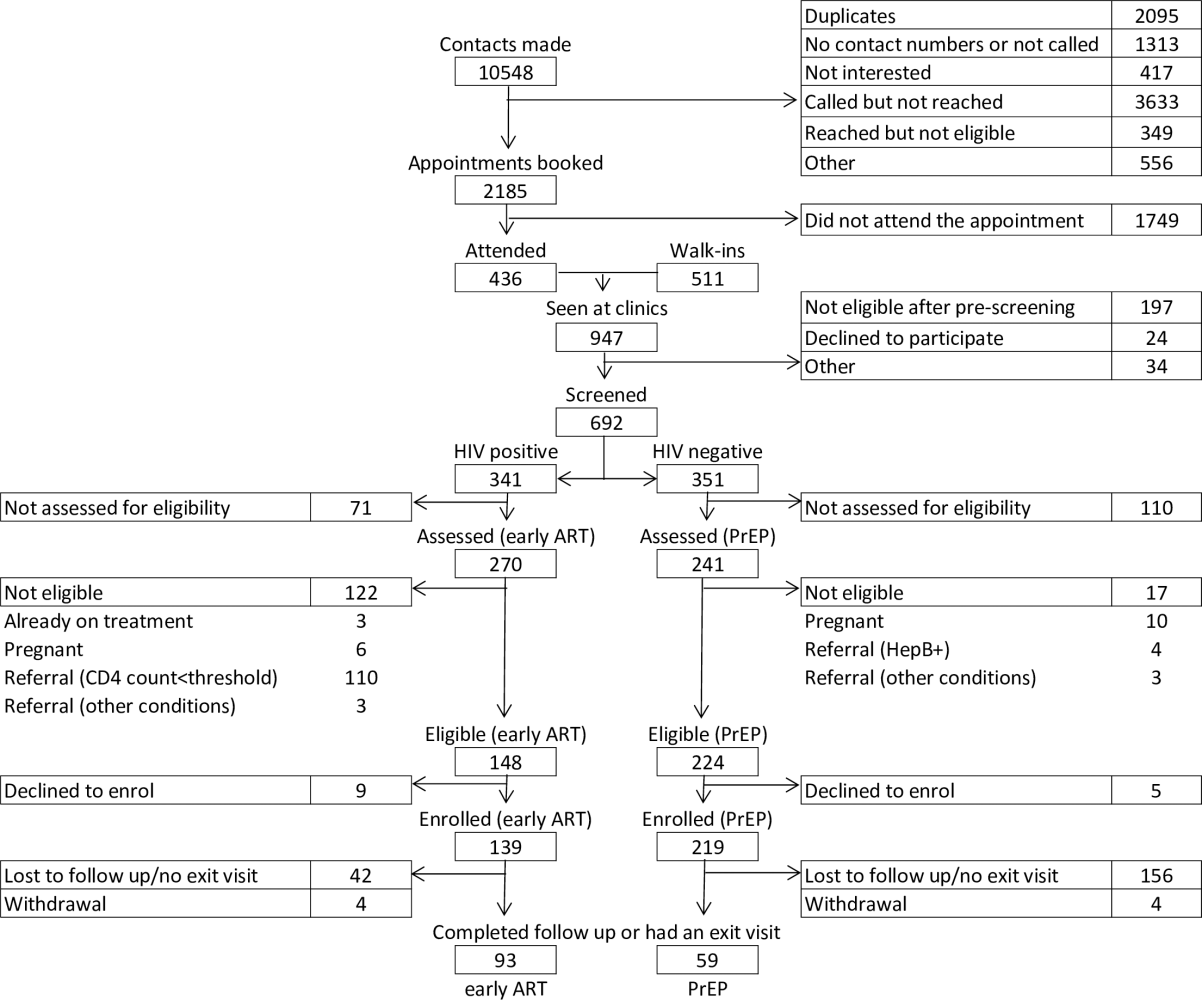
Flowchart of participant enrolment and follow-up. ART, antiretroviral treatment; HepB+, hepatitis B positive; CD4 count<threshold, CD4 count results below the threshold for ART initiation at the time of assessment; PrEP, pre-exposure prophylaxis.

Enrolment for the TAPS study occurred between 30 March 2015 and 31 July 2016. The process began with outreach and recruitment, by which 7,140 FSWs were contacted of the 10,548 names gathered across the 2 sites, after excluding duplicates and inaccurate phone numbers (Fig 1). Contacts included women recorded on waiting lists leading up to the launch of the study that were compiled during pre-study outreach and community education activities. After making contact, a total of 947 FSWs were seen in the clinic, of which 692 were HIV tested (73%) as per standard of care. At this point, 21% (*n =* 197) of the women were found to be already taking ART, to be pregnant, or to not consider themselves to be sex workers, making them ineligible for enrolment. These women were referred to relevant care options, and therefore did not complete the next phase of study screening, which occurred in the same visit as the HIV testing. Among those tested for HIV, there was a prevalence of 49% (*n =* 341/692). Following HIV testing, 79% (*n =* 241/351) and 69% (*n =* 270/341) participants returned for enrolment for PrEP and early ART, respectively. Among the HIV-negative participants who returned for eligibility assessment, 93% were assessed as clinically eligible for PrEP (*n =* 224/241). Among the HIV-positive participants who returned for eligibility assessment, 41% (*n =* 110/270) had CD4 counts within NDoH ART initiation guidelines at assessment, and 93% of those remaining were eligible for early ART (*n =* 148/160). Reasons for non-eligibility are presented in Fig 1. Uptake among those eligible was high: 98% (*n =* 219/224) of FSWs offered PrEP accepted, and 94% (*n =* 139/148) of FSWs offered early ART accepted. At the end of this process, 219 HIV-negative and 139 HIV-positive FSWs were enrolled. The detailed numbers of participants at each step of enrolment and follow-up are presented in Table A of S3 Text.

The comparison of TAPS cohorts, both PrEP and early ART, disaggregated by time of enrolment (e.g., before or after the launch of the national sex worker HIV programme) revealed no statistically significant differences throughout the enrolment and retention cascade, aside from 2 points: number of appointments booked (20% of contacts made resulted in an appointment being booked in the cohort enrolled before the national programme as opposed to 26% among those enrolled after the programme launch) and number eligible for early ART (more HIV-positive participants were eligible for early ART in the cohort recruited after the programme launch, 95%, compared to the cohort enrolled before the programme launch, 51%). In particular, the higher proportion of appointments booked from contacts made among those enrolled in the cohort after the launch of the programme compared to those enrolled before the programme could be related to larger recruitment and education efforts at the sites. The higher proportion of participants eligible for early ART in the cohort recruited after the programme started could be an indication of earlier presentation. The comparison of TAPS cohorts, both PrEP and early ART, disaggregated by completion of follow-up period (e.g., 12-month follow-up completed before or after the launch of the national programme) revealed no statistically significant differences throughout the enrolment and retention cascade. These results are presented in Table B of S3 Text. Further details regarding enrolment statistics over time can be found in Fig C of S3 Text.

### Baseline characteristics

In Table 1, selected baseline characteristics of enrolled participants are presented. Women enrolled in the PrEP group were significantly younger than those enrolled in the early ART group. In both groups, a substantial fraction of FSWs were currently in a steady partnership (48% on early ART and 53% on PrEP), were born in Zimbabwe (48% on early ART and 67% on PrEP), and had a secondary education (86% on early ART and 87% on PrEP). A substantial majority of the participants worked in brothels: 62% in the early ART group and 77% in the PrEP group.

**Table 1:** Baseline characteristics of female sex worker participants enrolled (total *n =* 358).

Characteristic	Detail	Early ART (*n =* 139)		PrEP (*n =* 219)		*p-*Value*
		*n*	Percent, mean, or median	*n*	Percent, mean, or median	
**Age**	**Mean (SD)**	139	31.9 (6.4)	219	29.8 (5.9)	0.0016
	**Median (min–max)**		31.7 (19.6–40.0)		28.9 (18.0–55.4)	
	**Age group**					
	18–20 years	1	0.7%	2	0.9%	0.001
	21–30 years	53	38.1%	127	58.0%	
	31–40 years	71	51.1%	77	35.2%	
	41–50 years	14	10.1%	10	4.6%	
	51–60 years	0	—	3	1.4%	
**Current partnership**	**No current partner**	70	50.4%	102	46.6%	0.599
	Single, no partners at present	64	46.0%	88	40.2%	
	Divorced or separated	5	3.6%	7	3.2%	
	Married and living apart	1	0.7%	7	3.2%	
	**Currently in a steady partnership**	67	48.2%	116	53.0%	
	Steady partner, not married/not living together	52	37.4%	85	38.8%	
	Steady partner, not married but living together	14	10.1%	24	11.0%	
	Married and living together	1	0.7%	7	3.2%	
	**Did not answer**	2	1.4%	1	0.5%	
**Country of origin**	Zimbabwe	67	48.2%	146	66.7%	0.009
	South Africa	60	43.2%	60	27.4%	
	Lesotho	7	5.0%	10	4.6%	
	Swaziland	3	2.2%	1	0.5%	
	Mozambique	2	1.4%	2	0.9%	
**Education**	No education	0	0.0%	1	0.5%	0.107
	Primary	15	10.8%	12	5.5%	
	Secondary	120	86.3%	189	86.3%	
	Tertiary	4	2.9%	15	6.9%	
	Did not answer/does not know	0	0.0%	2	0.9%	
**Place of work**	Hotel/brothel	86	61.9%	168	76.7%	0.008
	Street	37	26.6%	28	12.8%	
	Home	4	2.9%	5	2.3%	
	Other	12	8.6%	18	8.2%	

*All *p*-values are 2 sided; differences in means were assessed using *t* tests, and categorical variables were assessed using chi-squared tests.

ART, antiretroviral treatment; min, minimum; max, maximum; PrEP, pre-exposure prophylaxis.

### Visit attendance and retention

**Fig 2:**
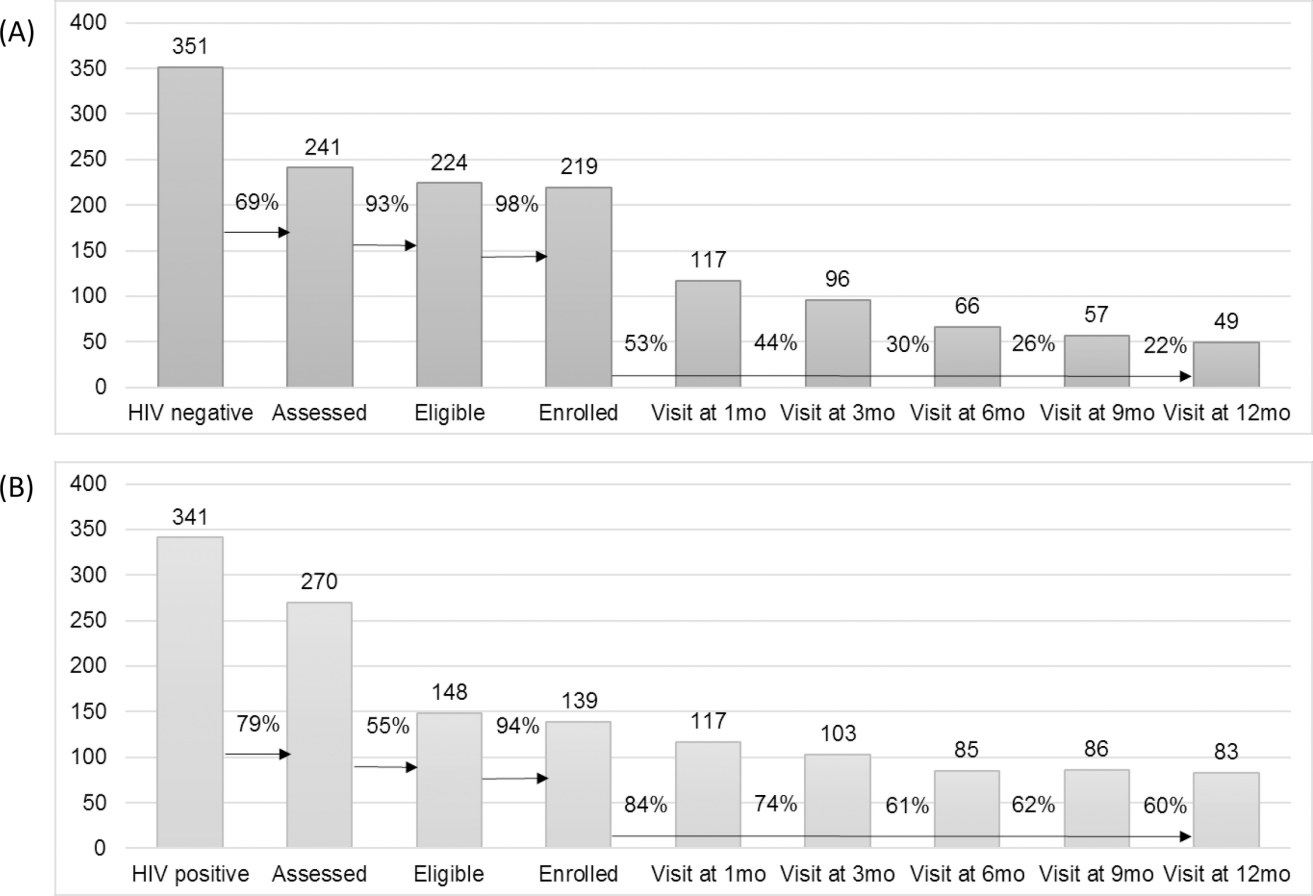
HIV prevention and care cascades. (A) HIV prevention cascade: pre-exposure prophylaxis cohort. (B) HIV treatment cascade: early antiretroviral treatment cohort.

During the first 12 months of follow-up, 156 and 42 participants (71% and 30%) were lost to follow-up (missed 2 clinical monitoring visits or did not complete an exit visit at 12 months) from the PrEP and early ART groups, respectively. The prevention and care cascades are shown in Fig 2.

The study follow-up period closed in 30 June 2017. In all, 22% and 60% of the enrolled PrEP and early ART cohorts, respectively, attended the 12-month visit. Overall, programme adherence was higher in the early ART group compared to the PrEP group, where participants tended to be less regular in their attendance. This is illustrated graphically in Fig D of S3 Text. Eight women withdrew, 4 from each group. Reasons for withdrawal were either side effects or moving to another location. Extended cascade results for those women who attended the study for a period of time longer than 12 months are presented in Table E of S3 Text. When examining loss to follow-up over time, as shown in Fig F of S3 Text, we observed that most women dropped off earlier in the schedule, in particular in the PrEP group. Denominators for each point in the cascade are also shown in Table E of S3 Text and should be taken into account for the results regarding loss to follow-up over time shown in Fig F of S3 Text.

### Secondary outcomes

**Fig 3:**
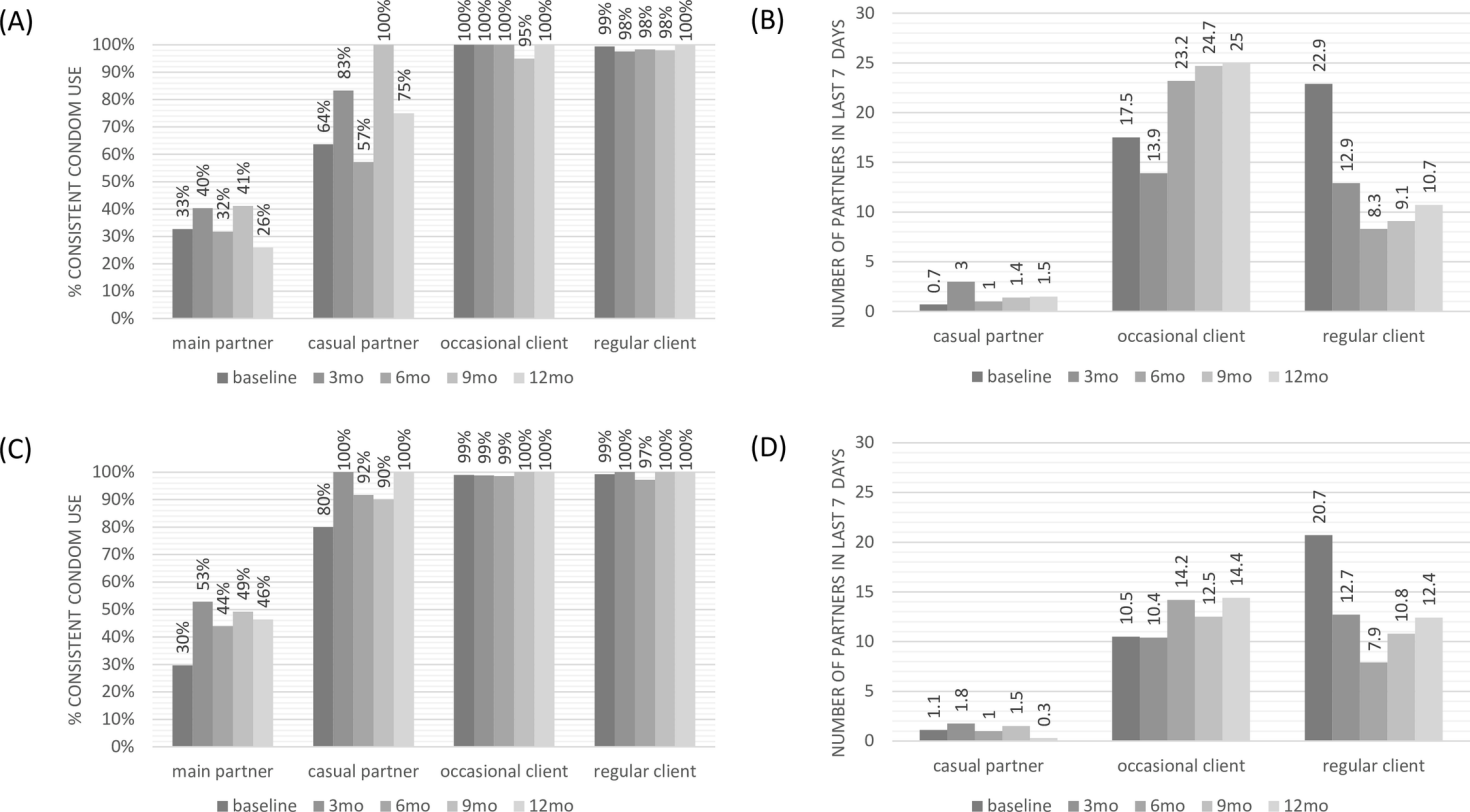
Sexual behaviour over time by partner type: consistent condom use and number of partners in last 7 days. (A) Consistent condom use: pre-exposure prophylaxis cohort. (B) Number of partners in the last 7 days: pre-exposure prophylaxis cohort. (C) Consistent condom use: early antiretroviral treatment cohort. (D) Number of partners in the last 7 days: early antiretroviral treatment cohort.

In Fig 3, self-reported consistent condom use by partner type and study group is shown over time. Higher condom use was reported with clients than with partners, especially main partners (steady partners). Women on early ART appeared to use condoms slightly more with casual and main partners than those on PrEP. Little change in reported condom use over time by type of partner was observed. Additionally, there was no apparent increase in number of partners over time. Further details on these data are presented in Table G in S3 Text. Number of STI episodes at baseline and every 3 months are presented in Table H in S3 Text. As compared with the number of episodes at study baseline, FSWs had significantly fewer STI episodes while participating in the study in both groups. These data are presented with the caveat that dropout over time may contribute to bias in the numbers at later time points.

The proportion of women reporting taking PrEP pills daily varied over time between 70% and 85%, whereas over 90% of participants reported taking pills daily while on early ART during the first 12 months of follow-up (Table I in S3 Text).

During the first 12 months of follow-up, there were 7 pregnancies in the PrEP group: Three women chose to terminate the pregnancy and continue on PrEP in the study, 3 were lost to follow-up, and 1 chose to continue on PrEP in the study while pregnant. There were 8 pregnancies in the early ART group, and an additional 4 in the extended follow-up period, 2 of which were identified at study exit. All early ART participants chose to continue with the study and their pregnancies.

One participant from the PrEP group who had previously decided to leave the study returned after she became HIV-positive and was referred for ART initiation. In the early ART group, there were 12 confirmed cases of virological failure—11 of which resulted in high-level virological resistance to at least 1 drug (11 with resistance to efavirenz, 5 to emtricitabine, and 2 to tenofovir)—among the women retained in the study. One patient had fully susceptible virus at resistance testing.

Of all 692 women who completed the screening process, only 1 participant presented with serious complicating health issues, including suspected TB and a creatinine clearance of 50 ml/min. There were no other cases presenting with creatinine clearance values outside of the threshold for initiation of tenofovir.

PrEP and treatment drugs were well tolerated, with the majority of adverse events being reported as mild for both groups, and few moderate. Of the 306 total adverse events recorded, only 17 were assessed to be drug related: 8 women on PrEP reported mild headaches, nausea, drowsiness, dizziness, or diarrhoea, and 9 women on early ART reported mild nausea, diarrhoea, dizziness, or drowsiness. One participant developed abnormal liver function tests, presenting ill after taking early ART for 4 months. Liver function tests revealed severely abnormal liver function, and the woman was referred to a specialist, where the diagnosis was HIV-induced sclerosing cholangitis.

Finally, in Table 2, both unit cost per visit type and overall cost of a person-year on early ART and PrEP are shown. We found lower unit costs for the visit types involving low overheads, few drugs or tests (i.e., outreach visits), or less staff time (i.e., refill visits). The distribution of costs by input is presented in Fig J of S3 Text.

**Table 2:** Unit costs per visit and per person-year for PrEP and early ART among female sex workers (2015 US dollars).

Unit cost	Site		Mean
	Johannesburg	Pretoria	
Outreach contact	3.0	2.6	2.8
VCT session	21.2	15.1	18.1
PrEP enrolment visit	40.4	29.0	34.7
PrEP monitoring visit	37.4	33.0	35.2
PrEP refill visit	7.4	6.2	6.8
**PrEP per person-year, y1**	**146.6**	**106.6**	**126.6**
Early ART enrolment visit	67.1	64.0	65.5
Early ART monitoring visit	72.5	62.9	67.7
Early ART refill visit	13.4	9.8	11.6
**Early ART per person-year, y1**	**380.5**	**432.3**	**406.4**

ART, antiretroviral treatment; PrEP, pre-exposure prophylaxis; VCT, voluntary counselling and testing; y1, initial year of services.

## Discussion

In this prospective observational demonstration project, we observed high uptake of the PrEP and early ART interventions among eligible FSWs. Retention in the early ART group was consistent with the national programme [21], while retention in the PrEP group was lower at the 12-month assessment. There were few virological failures in the early ART group and no seroconversions in the PrEP group. However, given the low rate of retention at the end of the study in the PrEP group, it is possible that women who dropped out and were not tracked became HIV-positive. To our knowledge, this is the first demonstration project to present results following the integration of a combined PrEP/early ART intervention into an existing sex worker health programme. The results of this project supported the establishment of the first official combined guidelines for oral PrEP and early ART in Africa, as well as the first specialised national programming including these interventions for sex workers in Africa [22,23]. The South African PrEP and early ART (or test and treat) guidelines were launched initially for sex workers in June 2016, at which point PrEP and initiation of ART irrespective of CD4 count became standard of care for sex workers. Test and treat was then extended to all HIV-positive people in South Africa in September 2016.

Additionally, TAPS has produced 2 important programmatic findings. First, the high number of women informed about the study translated into a much smaller number actually presenting to take up the interventions. Since both PrEP and early ART are novel interventions in this setting and for this population group, it is possible that as knowledge and use spread in this community, overall uptake might increase, as seen with the introduction of contraception [24]. Normalisation of new interventions may be key to their successful implementation [25], and this will be explored further in the analysis of qualitative data. Second, among those who presented at the clinic, there were high levels of intervention uptake for both PrEP and early ART.

The early ART group had a higher level of retention than the PrEP group. It is important to note that retention in care refers to 2 different constructs depending on whether participants are being retained in a prevention or treatment intervention. For the PrEP group, we formulated our definition of retention to reflect maintenance of continuous contact with healthcare services and access to prevention packages, even though the women were allowed to cycle in and out of PrEP medication use. For the early ART group, we valued retention in care as an essential component of a successful treatment programme. Interestingly, women who enrolled on PrEP earlier in the study tended to be the ones who continued the longest, suggesting that those most motivated, interested, and/or able to take up the interventions came earlier. It is important to note that participants were not reimbursed for their participation, suggesting that dedicated sex worker services can result in high levels of engagement in care among FSWs.

We observed a significant participant drop-off between screening and assessment for eligibility. Much of the loss may be directly related to the waiting period between screening and enrolment required for research purposes and to assess laboratory results. This may not reflect service delivery in future programmes where same-day initiation is being considered for both PrEP and early ART. We had only a single creatinine case at screening meriting concern, and immediate initiation of PrEP, with follow-up and potential discontinuation for those with concerning results, may be possible.

Women who became pregnant in either study group were given options regarding maintaining PrEP/ART use and staying in the study or transferring into other care. Of those who continued on PrEP in the study, 1 woman continued her pregnancy, while 3 terminated their pregnancies. It is possible that those who were lost to follow-up decided to see their pregnancies through, but this is unconfirmed. It is also possible that by excluding those who were pregnant at enrolment, women highly motivated to take PrEP were not given the opportunity. Given the heightened risk of HIV infection in women who become pregnant, being able to continue PrEP use during pregnancy may be a critical programming consideration.

At baseline, the TAPS cohorts had similar demographic characteristics (age, education, and place of origin) to the recently described local FSW population in Johannesburg [26]. Yet there was a higher rate of FSWs in stable relationships in our sample. This is likely a reflection of the group of FSWs accessing SWP clinics in general, as a similar profile was recently reported among SWP attendees not enrolled in the study [27].

The patterns of visits to the clinic for PrEP combined with the reported adherence would suggest intermittent use of PrEP while maintaining an overall engagement with services. Although we only analysed self-reported adherence data in this paper, the data were collected at every clinic and pill collection visit, and women did not report perfect adherence and usually reported when they took ‘PrEP breaks’, suggesting a low level of social desirability bias. Additionally, with no seroconversions among the PrEP users, intermittent PrEP use does not appear to be an issue in maintaining negative HIV status, noting also the high reported use of condoms with clients. Reassuringly, there was no observed change over time in the study in reported consistent condom use or number of partners in the last 7 days. There were fewer STI episodes during the study than at baseline for both groups. This finding, combined with the results of rigorous and consistent data collection on condom use over time with all sexual partners, suggests that the women who remained in the study may have been improving their attention to prevention through engagement in the study. This finding makes for an interesting comparison with another completed demonstration project study among MSM, where moderate increases in STIs during the study suggested slight reductions in condom use [28].

Finally, we estimated the total costs for PrEP and early ART per person-year to be in the same order of magnitude as recently published estimates [29,30]. The higher TAPS costs for early ART compared to the costs included in the South African HIV and TB Investment Case reflect the TAPS programme procedures, service utilisation, and staff costs, which are likely higher than the costs of routine services [30].

### Limitations

One limitation of the study is that no comparison group was included for either intervention, limiting the ability to assess the effectiveness of integrated services, as it would not have been ethical to maintain comparison groups with no or delayed access to interventions shown to be effective [31]. A second limitation is that efforts to replicate healthcare delivery through an integrated service were affected both by regulatory (dispensing from a pharmacy and medical officer assessment of adverse events) and ethics committee (informed consent administration) requirements. However, we expect that the removal of these requirements would improve rates of uptake and retention.

In addition, a national PrEP and early ART programme for FSWs in South Africa started 1 June 2016. After its initiation, this programme could have influenced the uptake of the interventions for those FSWs approached for enrolment in TAPS. Conversely, it could have influenced retention within the programme for those FSWs for whom the follow-up period overlapped with the provision of the national programme. The TAPS clinics were the only providers of PrEP free of charge to FSWs in the public setting and required informed consent to access the interventions as part of a research study, until the rollout of the national programme, which also provided these services at no cost to clients. Furthermore, the implementation of PrEP as part of the national programme meant that PrEP could be viewed as the standard of care and was therefore more normalised from the population’s perspective. Since PrEP was no longer considered experimental after 31 May 2016, we expected that women would think differently about PrEP, which could have affected uptake and retention in TAPS. For example, as the programme was scaled up in the initial 11 sites (including in the 2 TAPS sites), we became aware that some of the TAPS participants opted to collect their PrEP from the mobile clinics in Pretoria, rather than coming to the fixed clinic where TAPS was located.

Finally, we do not have data on the women who did not come back to the study, and the adherence and sexual behaviour data are self-reported, increasing the possibility of bias. Unfortunately, drug level analyses in the PrEP group were not available at the time of writing. These analyses will be presented in a more in-depth analysis in a subsequent publication. Additionally, the data reported are based on populations of FSWs in 2 specific urban settings, possibly limiting generalisability.

## Conclusion

The findings from TAPS informed the setup of the national programme of PrEP and test and treat for sex workers. This analysis shows that PrEP and early ART can be aligned with existing health service programming for FSWs safely, without significant behaviour change, with high rates of uptake for both interventions, and with expected cost reduction in routine settings at scale.

## Supporting information

S1 Fig TAPS project timeline. (PDF)

S1 Text STROBE statement for reporting of cohort studies. (DOCX)

S2 Text Costing methods. Table A. Data collection activities and sources of resource costs. Table B. Services and activities included by type of visit.
(PDF)

S3 Text Additional results. Table A. Detailed outreach, uptake, and retention statistics. Table B. Disaggregated outreach, uptake, and retention by enrolment and follow-up periods. Fig C. Distribution of enrolment over time and by site for PrEP and early ART. Fig D. Retention in PrEP and early ART programme (12-month follow-up). Table E. Extended HIV prevention and treatment cascades. Fig F. Participants last seen at clinic. Table G. Sexual behaviour over time by partner type: consistent condom use and number of partners in last 7 days. Table H. Sexually transmitted infections: episodes over time. Table I. Self-reported adherence over time (percent of participants reporting taking medication every day). Fig J. Mean cost by input and percentage of total mean unit cost by service (2015 US dollars).
(PDF)

## Abbreviations

ART: antiretroviral treatment
FSW: female sex worker
MSM: men who have sex with men
NDoH: National Department of Health
PrEP: pre-exposure prophylaxis
STI: sexually transmitted infection
SWP: Sex Worker Programme
TB: tuberculosis
