# Supplementary material for: HIV pre-exposure prophylaxis and early antiretroviral treatment among female sex workers in South Africa: Results from a prospective observational demonstration project
Source: PLoS Med. 2017 Nov 21;14(11):e1002444. doi: 10.1371/journal.pmed.1002444 (PMC5697804; doi:10.1371/journal.pmed.1002444)
Supplement: S1 Fig — (PDF) [file pmed.1002444.s001.pdf]

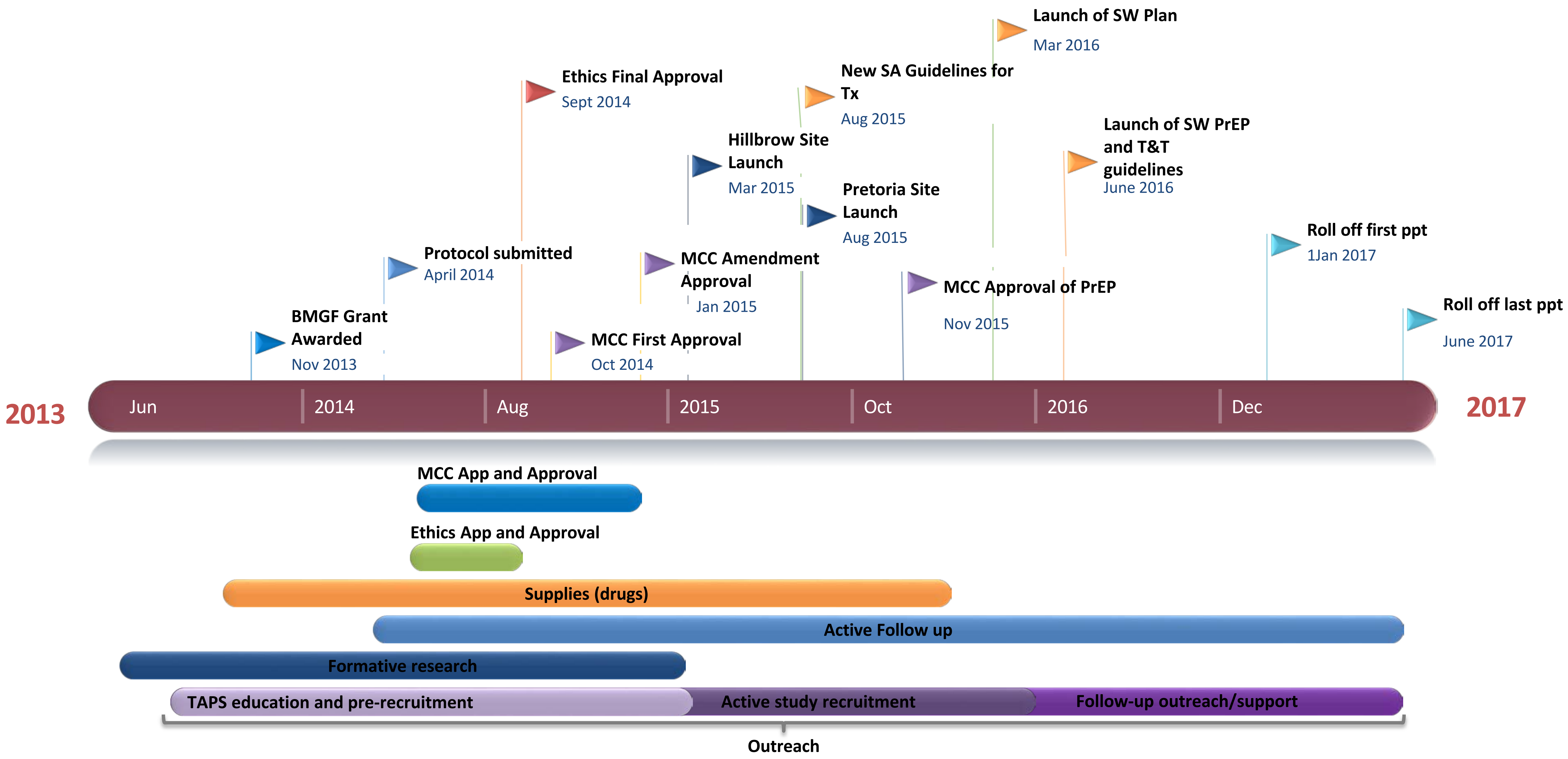

Abbreviations: Bill and Melinda Gates Foundation (BMGF); Medicines Control Council (MCC); South Africa (SA); HIV Treatment (Tx); Sex Worker (SW); Test & Treat (T&T); Participant (ppt)
