# Supplementary material for: HIV pre-exposure prophylaxis and early antiretroviral treatment among female sex workers in South Africa: Results from a prospective observational demonstration project
Source: PLoS Med. 2017 Nov 21;14(11):e1002444. doi: 10.1371/journal.pmed.1002444 (PMC5697804; doi:10.1371/journal.pmed.1002444)
Supplement: S2 Text — Table A. Data collection activities and sources of resource costs. Table B. Services and activities included by type of visit. (PDF) [file pmed.1002444.s003.pdf]

Costing methods

HIV pre-exposure prophylaxis and early antiretroviral treatment among female sex workers in South Africa: results from a prospective observational demonstration project

Eakle, R*; Gomez, GB*; Naicker, N; Bothma, R; Mbogua, J; Cabrera Escobar, MA; Saayman, E, M; Moorhouse, M; Venter, WDF; Rees, H on behalf of the TAPS Demonstration Project Team (*contributed equally)

Table A. Data collection activities and sources of resource costs.

	Site			
	Hillbrow	Pretoria	Total	Dates
Timesheets	13	12	25	October-November 2015
Observations (time montion data)	20	3	23	October-November 2015 & April-May 2016
Interviews with staff	14	13	27	October-November 2015 & April-May 2016
Expenditures review			2	October-November 2015 & April-May 2016
Utilisation data review			2	October-November 2015 & April-May 2017
Facility measurements (building, equipment, furniture)			1	October-November 2015

Resource	Source of information
Building	Facility measurements, floor plans, market rental prices
Equipment/Furniture	Ingredients approach: facility measurements and market replacement costs
HR	Interviews, time sheets, observations (time motion data), salaries and allowances obtained from human resource records
Drugs	Ingredients approach: drug prescription data from pharmacy and drug price from 2013 Department of Health tender price
Tests	Ingredients approach: facility records and test prices from NHLS 2015 price list and project invoices
Overheads	Facility and central project expenditure records for electricity, water, telephone and IT services (where applicable), security services, cleaning services, building and equipment maintenance, and waste removal (bio-safety and other)
Training (recurrent only)	Key staff interviews, health facility staff training records, expenditure records

In cases where resources were shared between research, service delivery and other services, we used the following allocation methods to exclude research and other services costs from our estimates: building space was allocated according to service distribution within the clinic's rooms and time used (from staff interviews and observations). Equipment and furniture use were allocated proportionally to the time used and number of participants (from staff interviews, observations and clinic records); staff time was allocated following observations of practices, timesheets, and staff interviews.

Table B. Services and activities included by type of visit.

Type of visit		Description	Provider
Outreach		Visits of sex work locations to distribute condoms and provide information on PrEP and early treatment services provided at With RHI's participating sites. Interested FSWs are given the opportunity to provide a phone number to be called for an appointment.	Team of CHW, peer educators (and occasionally nurse).
VCT		Includes obtaining VCT consent, re-testing counselling, testing and post-testing counselling.	CHW and nurses provide these services.
PrEP	enrolment	Clinical screening for PrEP initiation includes: creatinine clearance, hepatitis B surface antigen, urine pregnancy test, RPR, and syndromic STI screening.	Nurses perform the clinical procedures, if any complication a doctor is consulted.
	monitoring	Confirmation of HIV negative results at month 1 then VCT every three months, side effects, creatinine clearance, STI screening, behavioural sexual risk reduction counselling, adherence counselling and PrEP medication issuance.	Nurses perform the clinical procedures, if any complication a doctor is consulted. Pharmacist issues medication. Nurses or CHW provide counselling.
	refill only	Adherence counselling and PrEP medication issuance.	Pharmacist issues medication.
Early ART	enrolment	Early treatment initiation includes: ELISA confirmation of HIV status, CD4 cell count, creatinine clearance, hepatitis B surface antigen, urine pregnancy test, RPR, and syndromic STI screening.	Nurses perform the clinical procedures, if any complication a doctor is consulted.
	monitoring	Plasma viral load at 6 months, 12 months and then yearly; CD4 cell count at 12 months and then yearly; creatinine clearance at 3, 6, 12 months and then yearly; STI screening, behavioural sexual risk reduction counselling, adherence counselling and ART medication issuance.	Nurses perform the clinical procedures, if any complication a doctor is consulted. Pharmacist issues medication. Nurses or CHW provide counselling.
	refill only	Adherence counselling and ART medication issuance.	Pharmacist issues medication.
