# Supplementary material for: HIV pre-exposure prophylaxis and early antiretroviral treatment among female sex workers in South Africa: Results from a prospective observational demonstration project
Source: PLoS Med. 2017 Nov 21;14(11):e1002444. doi: 10.1371/journal.pmed.1002444 (PMC5697804; doi:10.1371/journal.pmed.1002444)
Supplement: S3 Text — Table A. Detailed outreach, uptake, and retention statistics. Table B. Disaggregated outreach, uptake, and retention by enrolment and follow-up periods. Fig C. Distribution of enrolment over time and by site for PrEP and early ART. Fig D. Retention in PrEP and early ART programme (12-month follow-up). Table E. Extended HIV prevention and treatment cascades. Fig F. Participants last seen at clinic. Table G. Sexual behaviour over time by partner type: consistent condom use and number of partners in last 7 days. Table H. Sexually transmitted infections: episodes over time. Table I. Self-reported adherence over time (percent of participants reporting taking medication every day). Fig J. Mean cost by input and percentage of total mean unit cost by service (2015 US dollars). (PDF) [file pmed.1002444.s004.pdf]

Additional results

HIV pre-exposure prophylaxis and early antiretroviral treatment among female sex workers in South Africa: results from a prospective observational demonstration project

Eakle, R*; Gomez, GB*; Naicker, N; Bothma, R; Mbogua, J; Cabrera Escobar, MA; Saayman, E, M; Moorhouse, M; Venter, WDF; Rees, H on behalf of the TAPS Demonstration Project Team (*contributed equally)

Table A. Detailed outreach, uptake, and retention statistics.

	All			
	n	N	%	[95% CI]
**ALL**				
**Contacts made**	10548			
**Appointment booked**	2185	10548	20,7%	[19.9-21.5]
**Seen at clinics**	947			
Attended	436	947	46,0%	[41.2-48.6]
Walk-ins	511	947	54,0%	[50.7-57.2]
**Screened**	692	947	73,1%	[70.1-75.9]
**PrEP**				
**HIV negative**	351	692	50,7%	[46.9-54.5]
**Assessed**	241	351	68,7%	[63.5-73.5]
**Eligible**	224	241	92,9%	[88.9-95.8]
**Enrolled**	219	224	97,8%	[94.9-99.2]
**Completed exit visit**	59	219	26,9%	[21.2-33.3]
Visit at 1mo	117	219	53,4%	[46.6-60.2]
Visit at 3mo	96	219	43,8%	[37.2-50.7]
Visit at 6mo	66	219	30,1%	[24.2-36.7]
Visit at 9mo	57	219	26,0%	[20.3-32.4]
Visit at 12mo	49	219	22,4%	[17.0-28.5]
**EARLY ART**				
**HIV positive**	341	692	49,3%	[45.5-53.1]
**Assessed**	270	341	79,2%	[74.5-83.4]
**Eligible**	148	270	54,8%	[48.7-60.9]
**Enrolled**	139	148	93,9%	[88.8-97.2]
**Completed exit visit**	93	139	66,9%	[58.4-74.6]
Visit at 1mo	117	139	84,2%	[77.0-89.8]
Visit at 3mo	103	139	74,1%	[65.9-81.2]
Visit at 6mo	85	139	61,2%	[52.5-69.3]
Visit at 9mo	86	139	61,9%	[53.3-70.0]
Visit at 12mo	83	139	59,7%	[51.1-67.9]

Table B. Disaggregated outreach, uptake, and retention by enrolment and follow up periods.

Influence of new National pilot on uptake was examined by disaggregating the cohort by time of enrolment according to when the national guidelines for PrEP and test and treat for sex workers was launched (1 June 2016). We also examined retention stratifying the cohort by follow up period (overlapping or not with the national pilot). We showed no major difference between cohorts. All p-values are two sided; differences in means were assessed using t-tests and categorical variables were assessed using chi square.

	**Difference in enrolment dates**							**Difference in 12-month follow up periods**						
	**Before 1June2016**			**From 1June2016**			**test 1**	**Completed before 1June2016**			**Completed from 1June2016**			**test 2**
	n	N	%	n	N	%	p	n	N	%	n	N	%	p
**ALL**														
**Contacts made**	9372			1176										
**Appointment booked**	1876	9372	20,0%	309	1176	26,3%	**<0.001**							
**Seen at clinics**	853			94										
Attended	378	853	44,3%	58	94	61,7%	**0.0009**							
Walk-ins	475	853	55,7%	36	94	38,3%	**0.0009**							
**Screened**	627	853	73,5%	65	94	69,1%	0.2972							
**PrEP**														
**HIV negative**	308	627	49,1%	43	65	66,2%	**0.0087**							
**Assessed**	211	308	68,5%	30	43	69,8%	0.8633							
**Eligible**	195	211	92,4%	29	30	96,7%	0.3270							
**Enrolled**	190	195	97,4%	29	29	100%	0.3446							
**Completed exit visit**	52	190	27,4%	7	29	24,1%	0.7091							
Visit at 1mo								92	172	53,5%	25	47	53,2%	1.0000
Visit at 3mo								50	120	41,7%	46	99	46,5%	0.4762
Visit at 6mo								25	98	25,5%	41	121	33,9%	0.2007
Visit at 9mo								13	48	27,1%	44	171	25,7%	0.8893
Visit at 12mo								0	4	0,0%	49	215	22,8%	0.2784
**EARLY ART**														
**HIV positive**	319	627	50,9%	22	65	33,8%	**0.0087**							
**Assessed**	248	319	77,7%	22	22	100%	**0.0128**							
**Eligible**	127	248	51,2%	21	22	95%	**0.0001**							
**Enrolled**	118	127	92,9%	21	21	100%	0.2077							
**Completed exit visit**	78	118	66,1%	15	21	71,4%	0.6344							
Visit at 1mo								85	103	82,5%	32	36	88,9%	0.3653
Visit at 3mo								47	66	71,2%	56	73	76,7%	0.4599
Visit at 6mo								37	59	62,7%	48	80	60,0%	0.7469
Visit at 9mo								28	45	62,2%	58	94	61,7%	1.0000
Visit at 12mo								8	12	66,7%	75	127	59,1%	0.6078

Figure C. Distribution of enrolment over time and by site on PrEP and early ART.

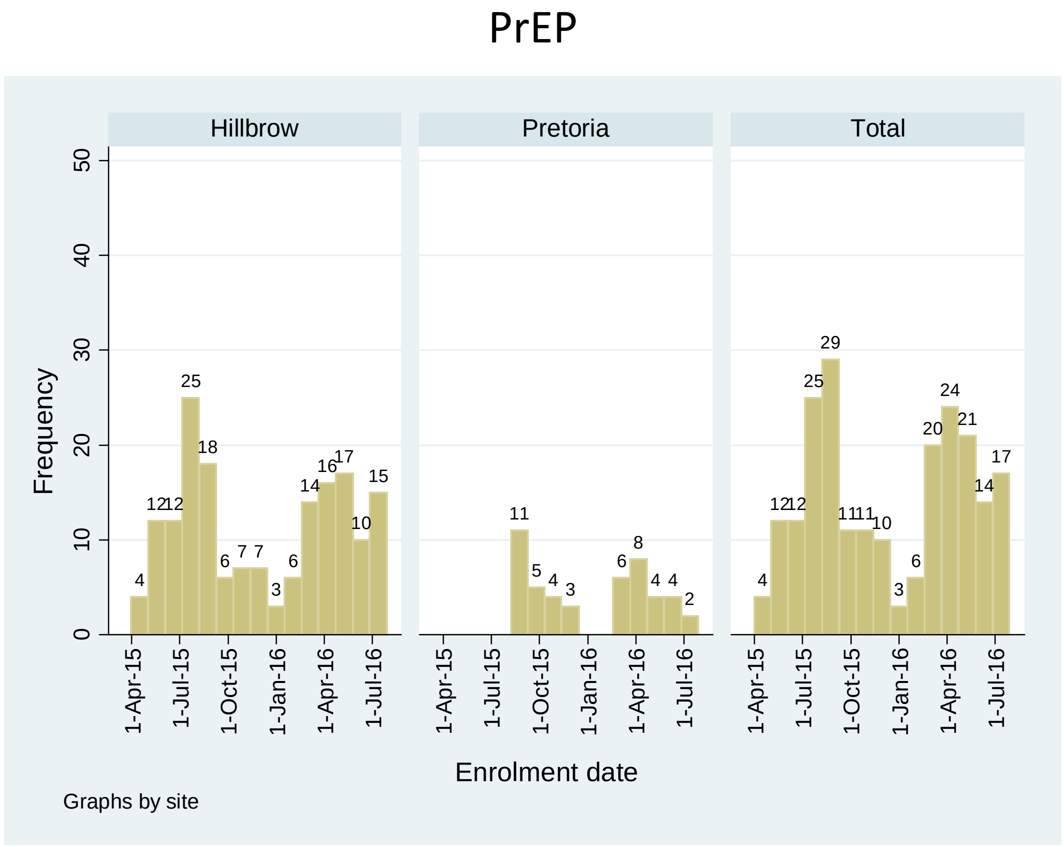

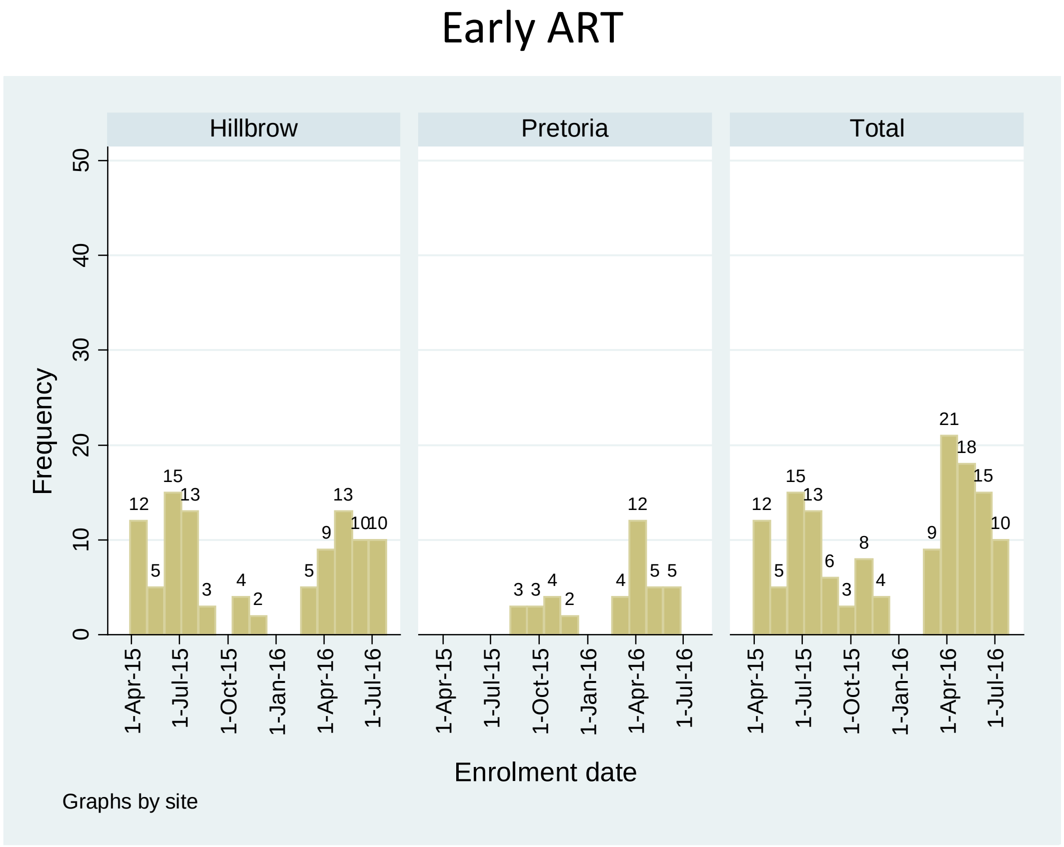

Figure D. Retention in PrEP and early ART programme (12-month follow up).

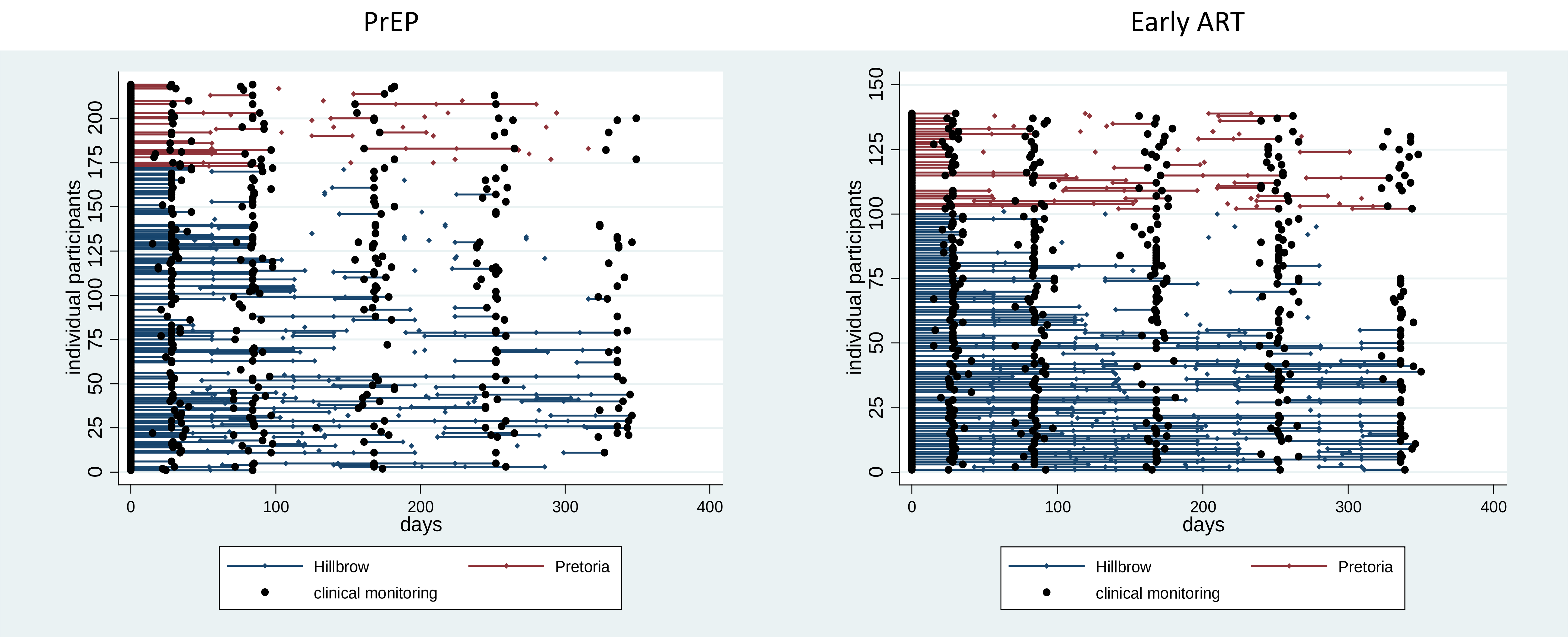

Individual participants are plotted in the y-axis; blue represents patterns of Hillbrow participants, while red is for Pretoria participants. Black dots were plotted for study visits, refills visits are represented in blue or red dots depending on the site. Continuous lines illustrate attendance to consecutive visits, and gaps in lines illustrate interruptions in the schedule. The pattern appears more continuous in the early treatment arm, while for PrEP, there is a more fluid or intermittent attendance pattern. Participants are sorted by recruitment date and number of visits, so that the lower part of the graph shows those participants recruited earlier in the programme.

Table E. Extended HIV prevention and treatment cascades.

	PrEP			Early ART		
	n	N	p [95% CI]	n	N	p [95% CI]
**Tested**	692			692		
**HIV positive**	-		-	341	692	0.49 [0.45-0.53]
**HIV negative**	351	692	0.51 [0.47-0.54]			-
**Assessed**	241	351	0.68 [0.63-0.73]	270	341	0.79 [0.75-0.83]
**Eligible**	224	241	0.93 [0.89-0.96]	148	270	0.55 [0.49-0.61]
**Enrolled**	219	224	0.98 [0.95-0.99]	139	148	0.91 [0.89-0.97]
**Visit at 1mo**	117	219	0.53 [0.47-0.60]	117	139	0.84 [0.77-0.90]
**Visit at 3mo**	96	219	0.44 [0.37-0.51]	103	139	0.74 [0.66-0.81]
**Visit at 6mo**	66	219	0.30 [0.24-0.37]	85	139	0.61 [0.53-0.69]
**Visit at 9mo**	57	219	0.26 [0.20-0.32]	86	139	0.62 [0.53-0.70]
**Visit at 12mo**	49	219	0.22 [0.17-0.29]	83	139	0.60 [0.51-0.68]
**Visit at 15mo**	38	172	0.22 [0.16-0.29]	54	103	0.52 [0.43-0.62]
**Visit at 18mo**	24	120	0.20 [0.13-0.28]	38	66	0.58 [0.45-0.70]
**Visit at 21mo**	21	98	0.21 [0.14-0.31]	39	59	0.66 [0.53-0.78]
**Visit at 24mo**	21	48	0.44 [0.29-0.59]	30	45	0.67 [0.51-0.80]

Figure F. participants last seen at clinic.

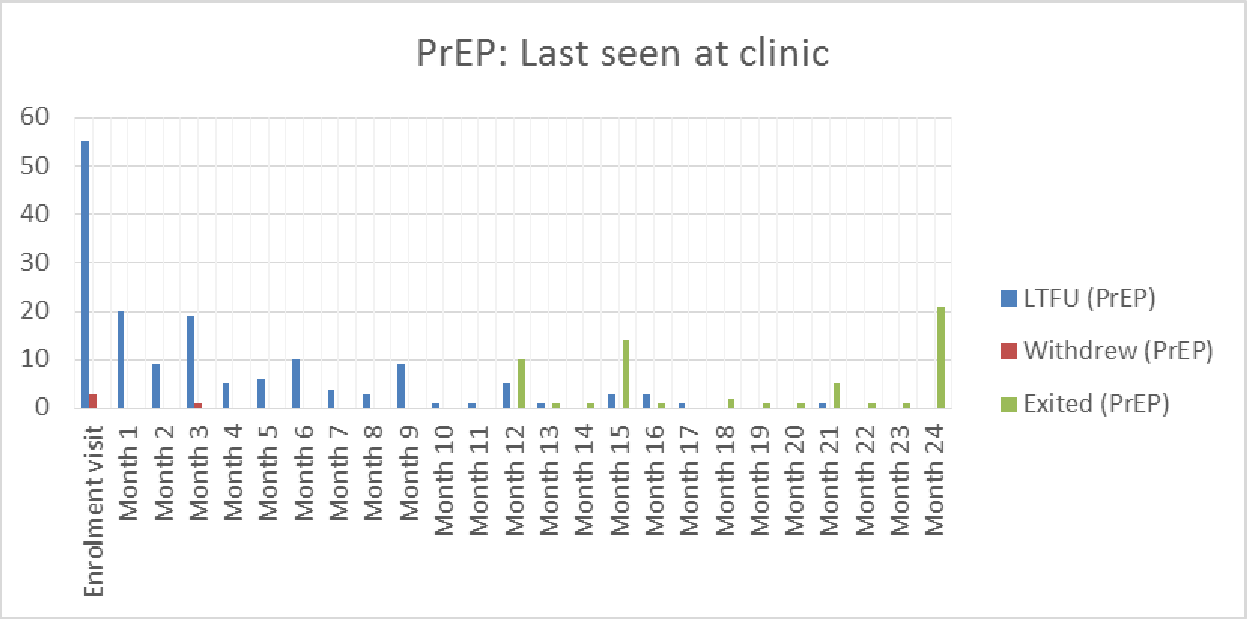

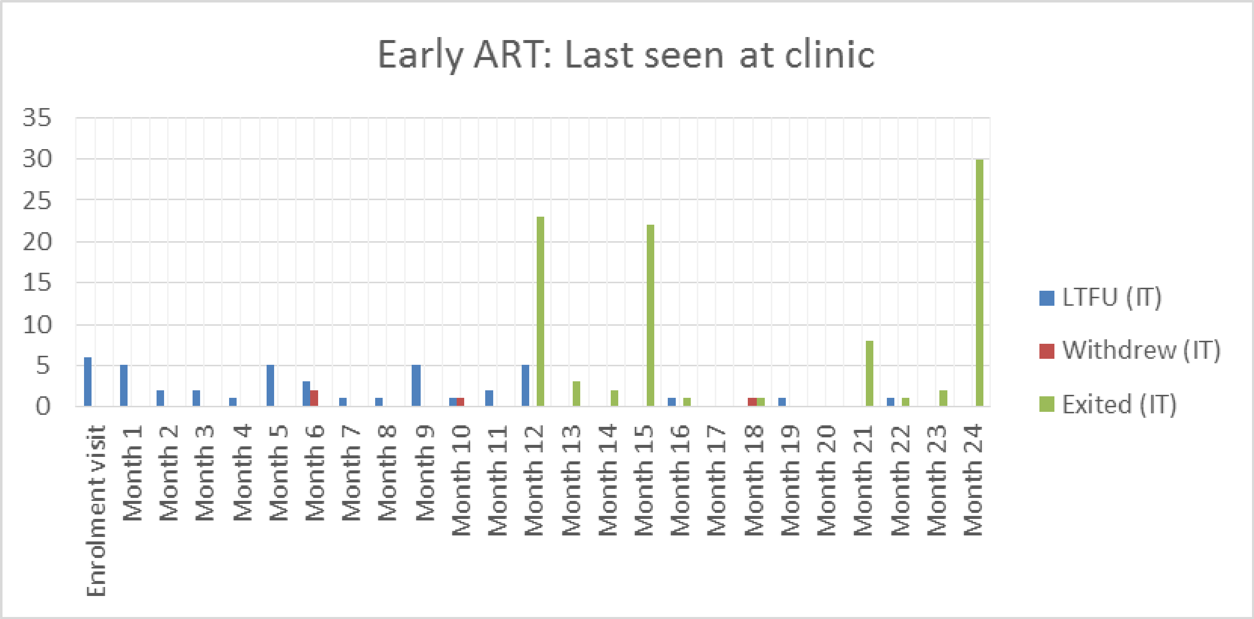

Definitions: LTFU = Lost to follow up (last point participant was seen at clinic and then lost six months after); Withdrew = participant withdrew herself; Exited = participant was transferred out at the end of her follow up to another clinic.

Table G. Sexual behaviour over time by partner type: consistent condom use and number of partners in last seven days.

Consistent condom use		baseline			3mo			6mo			9mo			12mo		
**Arm**	**partner type**	**n**	**N**	**%**	**n**	**N**	**%**	**n**	**N**	**%**	**n**	**N**	**%**	**n**	**N**	**%**
**PrEP**	main partner	47	144	33%	25	62	40%	13	41	32%	14	34	41%	7	27	26%
	casual partner	14	22	64%	5	6	83%	4	7	57%	5	5	100%	3	4	75%
	occasional client	181	181	100%	78	78	100%	54	54	100%	38	40	95%	33	33	100%
	regular client	179	180	99%	80	82	98%	58	59	98%	49	50	98%	31	31	100%
**Early ART**	main partner	27	91	30%	38	72	53%	29	66	44%	33	67	49%	19	41	46%
	casual partner	16	20	80%	12	12	100%	11	12	92%	9	10	90%	6	6	100%
	occasional client	100	101	99%	84	85	99%	68	69	99%	66	66	100%	45	45	100%
	regular client	124	125	99%	84	84	100%	69	71	97%	69	69	100%	45	45	100%

Number of partners		baseline		3mo		6mo		9mo		12mo	
**Arm**	**partner type**	**mean**	**SD**	**mean**	**SD**	**mean**	**SD**	**mean**	**SD**	**mean**	**SD**
**PrEP**	casual partner	0,7	1,1	3	3,2	1	1,5	1,4	1,1	1,5	1,3
	occasional client	17,5	21,8	13,9	13,2	23,2	32,4	24,7	31,9	25	22,1
	regular client	22,9	21,1	12,9	10,3	8,3	7,9	9,1	8,9	10,7	9,4
**Early ART**	casual partner	1,1	1,6	1,75	2,1	1	1,3	1,5	0,8	0,3	0,8
	occasional client	10,5	10,6	10,4	9,6	14,2	15,8	12,5	10,7	14,4	12,7
	regular client	20,7	20,1	12,7	9,9	7,9	7,4	10,8	10,1	12,4	8,5

Table H. Sexually transmitted infections: episodes over time.

	GUD	Genital warts	Herpes	Vaginal candidiasis	Vaginal discharge	Abscess	PID	TOTAL
PrEP								
**Baseline**	1	4	1	7	3	0	1	17
**month 3**	0	0	0	0	3	0	0	3
**month 6**	1	0	0	1	1	0	0	3
**month 9**	0	0	0	0	1	1	0	2
**month 12**	0	1	0	2	2	0	0	5
**month 15**	0	0	0	0	0	0	0	0
**month 18**	0	0	0	0	0	0	0	0
**month 21**	0	0	0	0	0	0	0	0
Early ART								
**Baseline**	1	5	4	6	7	3	3	29
**month 3**	0	0	1	0	4	1	0	6
**month 6**	0	0	0	3	0	1	1	5
**month 9**	0	0	0	1	1	1	0	3
**month 12**	0	0	0	0	2	0	0	2
**month 15**	1	0	0	0	0	0	0	1
**month 18**	0	0	0	1	1	0	0	2
**month 21**	0	0	0	0	0	0	0	0

Table I. Self reported adherence over time (% of participants reporting taking medication every day).

	n	N	%
PrEP			
**Baseline**	-	-	-
**month 3**	81	95	85%
**month 6**	52	66	79%
**month 9**	40	57	70%
**month 12**	32	38	84%
**month 15**	18	24	75%
**month 18**	21	22	95%
**month 21**	13	16	81%
Early ART			
**Baseline**	-	-	-
**month 3**	98	103	95%
**month 6**	77	84	92%
**month 9**	81	86	94%
**month 12**	56	60	93%
**month 15**	31	31	100%
**month 18**	35	36	97%
**month 21**	29	31	94%

Figure J. Mean cost by input and percentage of total mean unit cost by service (USD 2015).

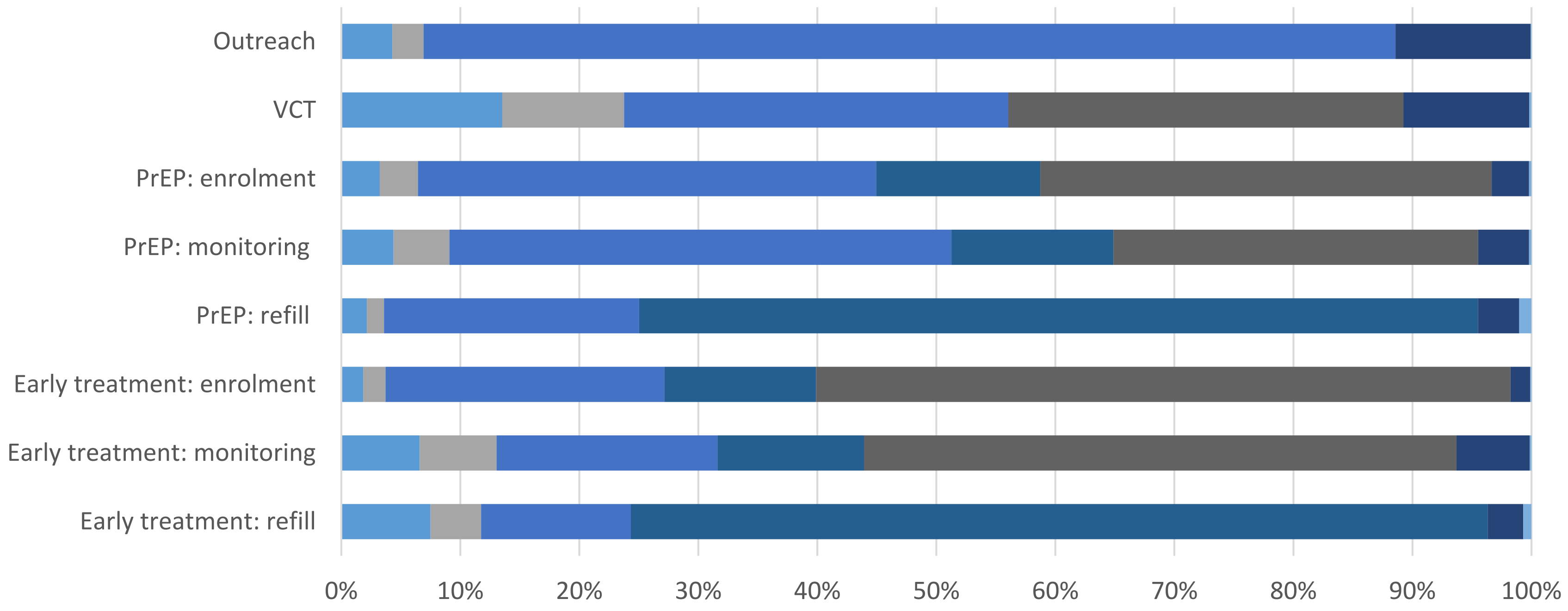

	Early treatment: refill	Early treatment: monitoring	Early treatment: enrolment	PrEP: refill	PrEP: monitoring	PrEP: enrolment	VCT	Outreach
Building	0,9	4,5	1,2	0,1	1,5	1,1	2,5	0,1
Equipment/Furniture	0,5	4,4	1,2	0,1	1,7	1,1	1,9	0,1
HR	1,5	12,6	15,4	1,5	14,8	13,4	5,9	2,3
Drugs	8,3	8,3	8,3	4,8	4,8	4,8	0,0	0,0
Tests	0,0	33,7	38,2	0,0	10,8	13,2	6,0	0,0
Overheads	0,3	4,2	1,1	0,2	1,5	1,1	1,9	0,3
Training	0,1	0,1	0,1	0,1	0,1	0,1	0,0	0,0
